# Toe keloid after nail extraction treated with surgical excision: A case report

**DOI:** 10.1097/MD.0000000000009373

**Published:** 2017-12-22

**Authors:** Hao Liu, Kexin Song, Mingzi Zhang, Xinhang Dong, Shu Liu, Youbin Wang

**Affiliations:** a Department of Plastic Surgery, Peking Union Medical College Hospital; b Department of Plastic Surgery, China Meitan General Hospital, Beijing, China.

**Keywords:** nail extraction, surgical excision, toe keloid

## Abstract

**Rationale:**

In this study, a case of toe keloid after nail extraction is presented, in which the keloids on both toes were resected by surgical excision. Keloids (from the Greek word meaning “crab's claw”) are fibrous growths that extend beyond the original area of injury to involve the adjacent normal skin. In general, keloid tendencies appear to be regionally isolated to keloid-prone areas, such as the chest, ears, and deltoid regions, whereas the hands and feet are usually spared, which is why this case is meaningful.

**Patient concerns:**

A 20-year-old Chinese man had paronychia on both halluxes when he was 16 years old. He underwent a nail extraction at the age of 17. The nails of both halluxes were removed by nail extraction. This operation was successful, and the postoperative course was uneventful. After 6 months, the scars of the nail extraction on both sides began to exhibit hyperplasia and became red and swollen with itching. Later, the scar expanded and eroded the tissue beyond the matrix unguis. The whole matrix unguis was destroyed, and the nails were distorted. The scars began to ulcerate after 2 years. The patient used potassium permanganate to clean his wounds, but the keloid scars did not improve.

**Diagnoses:**

The patient was diagnosed as toe keloid based on his history and symptoms. The biopsy result supported our diagnoses.

**Interventions:**

The toe keloids were effectively cured by surgical excision and skin flap transplantation combined with postoperative irradiation and hyperbaric oxygen (HBO) treatment.

**Outcomes:**

No recurrence was detected during the period from 6 to 24 months of follow-up after the surgery.

**Lessons:**

In this case, the trauma of the nail extraction was likely the key cause of the keloid. However, the patient was also predisposed to keloids, as we observed keloids on his chest. In general, keloid tendencies appear to be regionally isolated to keloid-prone areas such as the chest, ears, and deltoid regions, whereas the hands and feet are usually spared, which is why this case is meaningful.

## 1 Introduction

Keloids and hypertrophic scars represent an excessive tissue response to dermal injury characterized by local fibroblast proliferation and an overproduction of collagen.^[1,2]^ Keloids (from the Greek word meaning “crab's claw”) are fibrous growths that extend beyond the original area of injury to involve the adjacent normal skin.^[3]^ Keloids and hypertrophic scars may cause functional impairment and cosmetic disfigurement and are often associated with a self-reported low quality of life in patients.^[4]^ The most common keloid sites are the anterior chest, upper arm, earlobe, and shoulder. However, keloids located on the toes are rare.

Herein, we present a case of toe keloid that formed after the treatment of paronychia with nail extraction, which was effectively cured by surgical excision and a skin flap transplantation assisted by hyperbaric oxygen (HBO) treatment and postoperative irradiation. Our study was approved by the Ethics Committee of Peking Union Medical College Hospital, China. The use of all of the information and figures that are shown in this report was allowed by the patient.

## 2 Case report

A 20-year-old Chinese man had paronychia on both halluxes when he was 16 years old. He underwent a nail extraction at the age of 17. The nails of both halluxes were removed by nail extraction. This operation was successful, and the postoperative course was uneventful. After 6 months, the scars of the nail extraction on both sides began to exhibit hyperplasia and became red and swollen with itching. Later, the scar expanded and eroded the tissue beyond the matrix unguis. The whole matrix unguis was destroyed, and the nails were distorted. The scars began to ulcerate after 2 years. The patient used potassium permanganate to clean his wounds, but the keloid scars did not improve.

**Figure 1:**
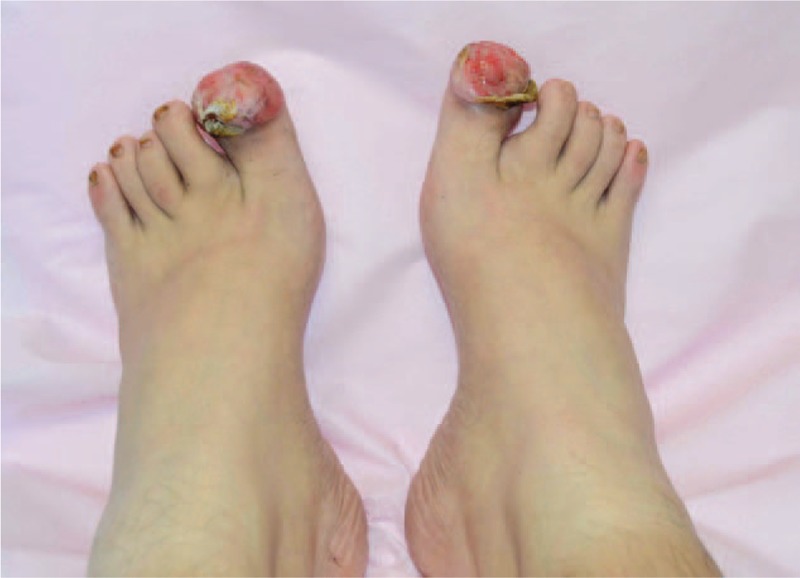
Toe keloids after nail extraction on both sides of the patient's feet.

This patient visited our outpatient facility in June 2015. The keloid tissue could be seen on both of his halluxes. The lesion on the left toe was 3 cm×4 cm × 3 cm, and the mass on the other toe was 3 cm × 3 cm × 4 cm, both of which had a red appearance and an irregular shape. The nails of both of his great toes were squeezed and distorted (Fig. 1). The patient complained of problems when walking and putting on shoes.

On the basis of the condition of this patient, surgical approaches were designed for both of his great toes. However, if the surgery was performed on both of his feet in 1 operation, the patient would have had problems walking for some time. Therefore, a 2-stage operation was planned to ensure a high quality of daily life. Each foot underwent the operation at an interval of 4 months. We performed the first stage on the left foot on September 9, 2015.

### 2.1 Surgical course

**Figure 2:**
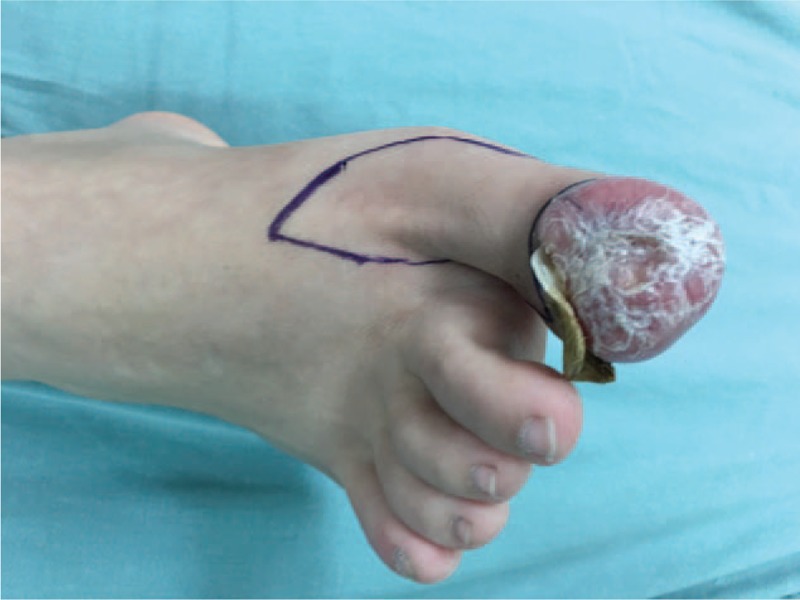
Designation of skin flap during the surgery.

**Figure 3:**
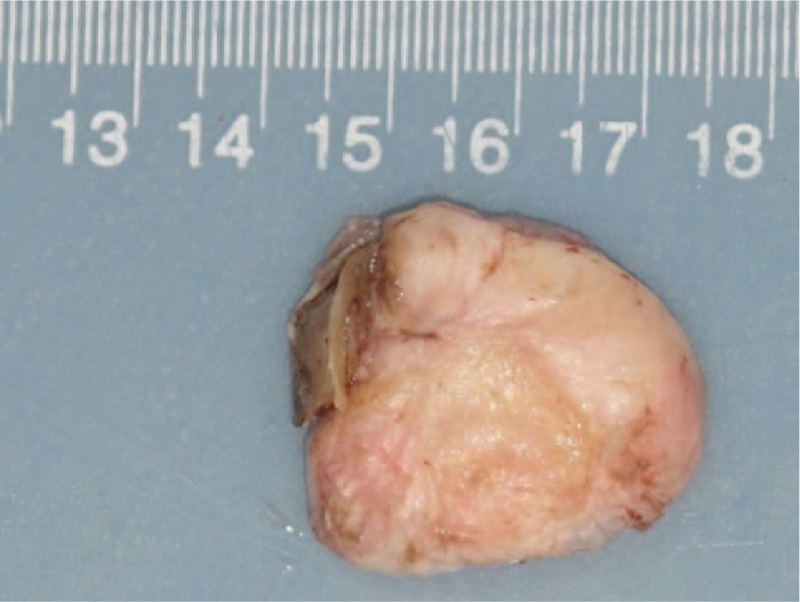
Mass removed from the right great toe. Approximately 3.1 cm × 2.6 cm.

**Figure 4:**
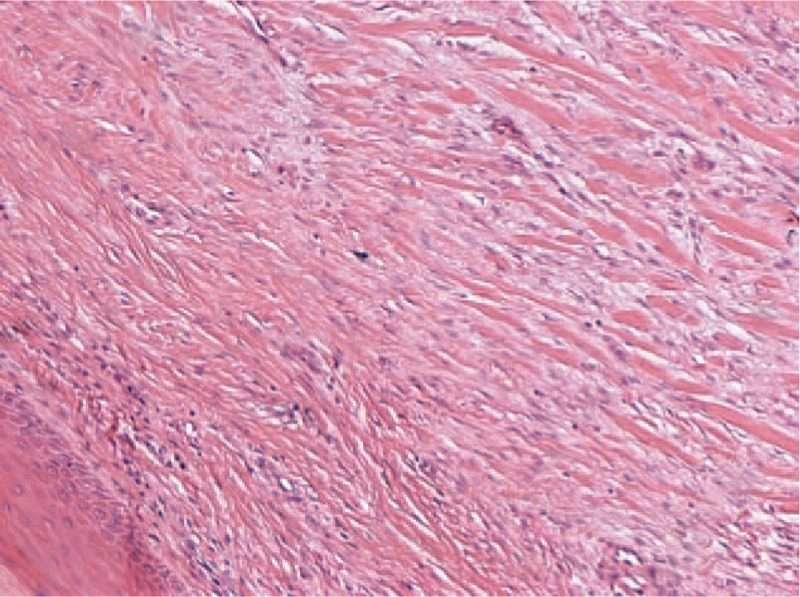
Hematoxylin and eosin staining. A biopsy specimen was diagnosed as a keloid under 10 × 40 lens.

After general anesthesia, a tourniquet was used to close the vessel once blood evacuation was performed. During the surgery, we loosened the tourniquet every hour to prevent necrosis of the tissue of the patient's foot. A skin flap with a vessel pedicle consisting of the dorsal digital artery of the hallux was designed (Fig. 2). The flap covered the dorsal skin of the hallux and some of the distal part of the foot. Then, the keloid was completely resected (Figs. 3 and 4). On the basis of the design, the skin flap was elevated, which was followed by a step-by-step bipolar coagulation. The flap was then advanced to cover the wound. Finally, the wound was closed with a 6–0 nylon suture.

### 2.2 Postoperative course

**Figure 5:**
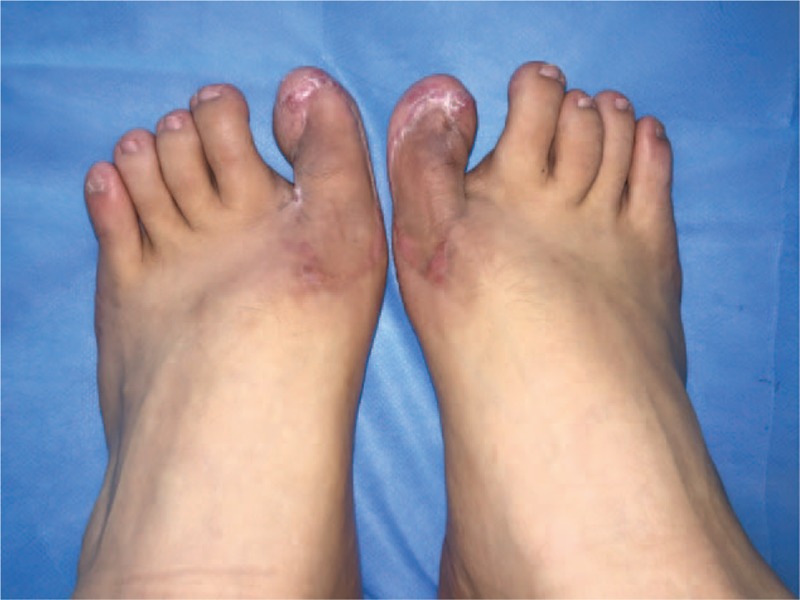
Toe appearance on both sides after keloid surgery (left: 12 mo; right: 6 mo). No recurrence has been detected.

**Figure 6:**
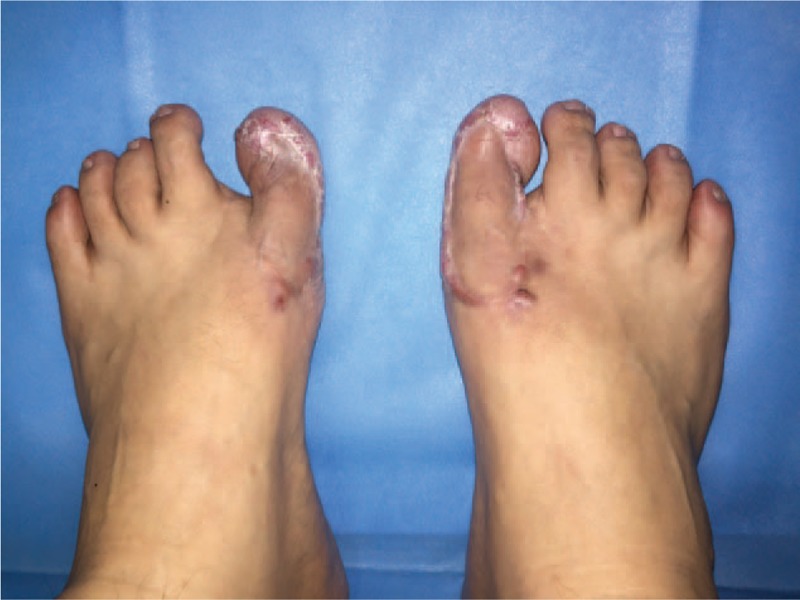
Toe appearance on both sides after keloid surgery (left: 24 mo; right: 18 mo). No recurrence has been detected.

After the operation, the patient received postoperative radiation twice at a depth of radiation of 1 cm and a radiotherapy dose of 900 Gy each time. The patient also received HBO therapy before the sutures were removed. The keloids of both great toes were cured in 6 months after 2 surgeries. No recurrence was detected during the period from 6 to 24 months of follow-up after the surgery (Figs. 5 and 6). The patient was quite satisfied with the result.

## 3 Discussion

We encountered a large keloid of the toe that was effectively cured with surgical treatment followed by postoperative irradiation and HBO treatment. A keloid is a fibro-proliferative disorder that invades the normal tissue and proliferates abnormally on the surrounding skin. Keloid formation is mainly caused by the abnormal proliferation of fibroblasts and the excessive formation and accumulation of extracellular matrixes during the healing process of scars. Such wounds have various causes, such as surgery, burns, trauma, inflammation, foreign body reactions, and endocrine dysfunctions.^[5]^ In this case, the trauma of the nail extraction was likely the key cause of the keloid. However, the patient was also predisposed to keloids, as we observed keloids on his chest.

In general, keloid tendencies appear to be regionally isolated to keloid-prone areas such as the chest, ears, and deltoid regions, whereas the hands and feet are usually spared,^[6]^ which is why this case is meaningful. Keloid formation on the toes and fingers is rare, and thus far, only 22 cases in 9 studies have been reported.^[7–15]^ Keloid formation after nail extraction is also quite rare. No previous report has identified a keloid caused by nail extraction. We also suggest that the operation method in this case is noteworthy because the operation result was reasonably good.

Various treatments for keloids have been described. In reports of 22 cases of toe and finger keloids, 12 cases reported using a skin graft to cover the wound after resecting the mass, 5 cases reported using a plantar-based rectangular flap to cover the wound, and the others reported closing the wound directly without flaps or skin grafts (Table 1). No reports have described using a local skin flap with a vessel pedicel consisting of the dorsal digital artery of the hallux to close the wound.

**Table 1:**
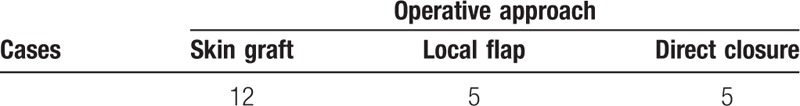
The cases of different operative approaches.

The hallux is supplied by the dorsal digital artery of the hallux and the plantar digital artery of the hallux. The dorsal digital artery of the hallux is the main blood supply of the dorsal skin. The subcutaneous tissue of the dorsal hallux skin is not rich, and a V-Y advancement local skin flap based on the subcutaneous tissue pedicle cannot be easily used because of the limited skin flap advancement and blood supply damage. On the basis of 1 side of the dorsal digital artery of the hallux, the skin flap can be clearly elevated, and the free side can be rotated and advanced easily to cover the wound on the distal part of the hallux without inducing blood supply damage. Furthermore, the appearance of the skin flap from the dorsal skin of the hallux will be more acceptable than skin grafts. In addition, the quality will also be better and more suitable to fulfill its task as toe skin, especially as the toe skin must be very durable considering the patient's life situation. The blood supply in the distal limb is not abundant, and wounds on the distal limb heal slowly and sometimes develop necrosis as a result of an inadequate blood flow. Therefore, we recommend that patients receive HBO treatment^[16]^ after the operation and postoperative irradiation.

## 4 Conclusion

We encountered a case of toe keloid formation after nail extraction that was effectively cured by surgical excision and skin flap transplantation combined with postoperative irradiation and HBO treatment.

## References

[1] Nemeth AJ Keloids and hypertrophic scars. J Dermatol Surg Oncol 1993;19:738–46. 8349914 10.1111/j.1524-4725.1993.tb00418.x

[2] Teofoli P Barduagni S Ribuffo M Expression of Bcl-2, p53, c-jun and c-fos protooncogenes in keloids and hypertrophic scars. J Dermatol Sci 1999;22:31–7. 10651227 10.1016/s0923-1811(99)00040-7

[3] Mahdavian Delavary B van der Veer WM Ferreira JA Niessen FB Formation of hypertrophic scars: evolution and susceptibility. J Plast Surg Hand Surg 2012;46:95–101. 22471257 10.3109/2000656X.2012.669184

[4] Bock O Schmid-Ott G Malewski P Quality of life of patients with keloid and hypertrophic scarring. Arch Dermatol Res 2006;297:433–8. 16528552 10.1007/s00403-006-0651-7

[5] Nicoletis C Bazin S Lous ML Clinical and biochemical features of normal, defective, and pathologic scars. Clin Plast Surg 1977;4:347–59. 884926

[6] Calnan JS Copenhagen HJ Autotransplantation of keloid in man. Br J Surg 1967;54:330–5. 4225729 10.1002/bjs.1800540504

[7] Yamawaki S Naitoh M Ishiko T A toe keloid after syndactyly release treated with surgical excision and intralesional steroid injection. J Plast Reconstr Surg Glob Open 2014;2:e186. 10.1097/GOX.0000000000000152 PMC4229290 25426369

[8] Wood VE Keloid formation in a simple syndactyly release: a case report. J Hand Surg Am 1992;17:479–80. 1319437 10.1016/0363-5023(92)90355-s

[9] Karpf AJ London E Rousso M Syndactylism with keloid scar formation. J Foot Ankle Surg 1993;32:509–13. 8252010

[10] Endo T Nakayama Y Uchida A Keloid formation after surgery for release of polysyndactyly of the feet in a child. Br J Plast Surg 1995;48:43–6. 7719608 10.1016/0007-1226(95)90030-6

[11] Smet LD Fabry G Keloid formation in syndactyly release: report of two cases. J Pediatr Orthop B 1997;6:68–9. 9039671 10.1097/01202412-199701000-00014

[12] Muzaffar AR Rafols F Masson J Keloid formation after syndactyly reconstruction: associated conditions, prevalence,;1; and preliminary report of a treatment method. J Hand Surg Am 2004;29:201–8. 15043889 10.1016/j.jhsa.2003.10.017

[13] Lim YJ Teoh LC Lee EH Reconstruction of syndactyly and polysyndactyly of the toes with a dorsal pentagonal island flap: a technique that allows primary skin closure without the use of skin grafting. J Foot Ankle Surg 2007;46:86–92. 17331867 10.1053/j.jfas.2006.11.008

[14] Tolerton SK Tonkin MA Keloid formation after syndactyly release in patients with associated macrodactyly: management with methotrexate therapy. J Hand Surg Eur Vol 2011;36:490–7. 21447529 10.1177/1753193411402146

[15] Kong BY Baek GH Gong HS Treatment of keloid formation following syndactyly division: surgical technique. Hand Surg 2012;17:433–7. 23061961 10.1142/S0218810412970088

[16] Wilkinson D Doolette D Hyperbaric oxygen treatment and survival from necrotizing soft tissue infection. Arch Surg 2004;139:1339–45. 15611459 10.1001/archsurg.139.12.1339

